# Estimating the Power of Indirect Comparisons: A Simulation Study

**DOI:** 10.1371/journal.pone.0016237

**Published:** 2011-01-21

**Authors:** Edward J. Mills, Isabella Ghement, Christopher O'Regan, Kristian Thorlund

**Affiliations:** 1 Faculty of Health Sciences, University of Ottawa, Ottawa, Canada; 2 Ghement Statistical Consulting Company, Richmond, Canada; 3 Department of Epidemiology, London School of Hygiene & Tropical Medicine, London, United Kingdom; 4 Department of Clinical Epidemiology & Biostatistics, McMaster University, Hamilton, Canada; University of Michigan, Canada

## Abstract

**Background:**

Indirect comparisons are becoming increasingly popular for evaluating medical treatments that have not been compared head-to-head in randomized clinical trials (RCTs). While indirect methods have grown in popularity and acceptance, little is known about the fragility of confidence interval estimations and hypothesis testing relying on this method.

**Methods:**

We present the findings of a simulation study that examined the fragility of indirect confidence interval estimation and hypothesis testing relying on the adjusted indirect method.

**Findings:**

Our results suggest that, for the settings considered in this study, indirect confidence interval estimation suffers from under-coverage while indirect hypothesis testing suffers from low power in the presence of moderate to large between-study heterogeneity. In addition, the risk of overestimation is large when the indirect comparison of interest relies on just one trial for one of the two direct comparisons.

**Interpretation:**

Indirect comparisons typically suffer from low power. The risk of imprecision is increased when comparisons are unbalanced.

## Introduction

In recent years, the adjusted indirect comparisons method, first suggested by Bucher et al.[1], has been widely used to compare competing treatments in the absence of direct evidence about their relative performance.[2] For instance, if two treatments B and C are compared against a common comparator, treatment A, via two distinct sets of randomized trials, this method can be used to derive an indirect estimate of the relative effect of B versus C on the basis of the direct estimates of the relative effects of B versus A and C versus A.

For the adjusted indirect method, it is generally well understood that the precision of the resulting indirect estimate of the relative effect of B versus C is lower than that of the direct estimate that would have been obtained if direct evidence from trials comparing B and C head-to-head were available.3 Indeed, under certain assumptions, it has been established that an indirect estimate of B versus C would have to be based, on average, on four times as many trials than a direct estimate to achieve the same precision as the direct estimate.[3] These assumptions are as follows: (i) all within-study variances are (approximately) equal within and across pair-wise comparisons of treatments, (ii) between-study variances are (approximately) equal across pair-wise comparisons of treatments and (iii) each pair-wise comparison of treatments includes an equal number of trials.

Wells et al.[4] have investigated in great detail the mean squared error properties of the indirect point estimation of the relative effect of B versus C by means of a simulation study. However, to our knowledge, there have been no attempts in the literature to expand the scope of this investigation to the study of the risk of overestimation as well as the properties of confidence interval estimation and hypothesis testing regarding the relative effect of B versus C.

The power of indirect comparisons to detect differences in treatment effects, if they exist, is a particularly important one for clinical practice. In settings where the direct evidence available for the comparison of B versus A is sparse relative to that available for the comparison of C versus A, we need to understand the extent to which the indirect comparison of B versus C may be under-powered. Intuitively, if the direct comparison of B versus A is under-powered, we would also expect the indirect comparison of B versus C to be under-powered, as it relies on the direct comparison of B versus A in addition to that of C versus A.

In this paper, we present the results of a simulation study that examines the performance of the following aspects concerning the indirect inference on the relative effect of B versus C: (i) overestimation associated with point estimation of the indirect estimate of B versus C (ii) coverage of confidence intervals for the relative effect of B versus C, (iii) type I error of tests of hypotheses concerning the relative effect of B versus C and (iv) power of tests of hypotheses concerning the relative effect of B versus C. Our study focuses on effects expressed on the odds ratio scale, though it could be easily extended to effects expressed on different scales.[4]

We start by explaining the Bucher method. We then describe the design of our simulation study and present its results. We conclude by discussing the practical implication of the findings of this simulation study.

### Adjusted indirect comparisons

In many situations, we are interested in assessing the relative effects of three different treatments – A, B and C – on the basis of randomized trials that have compared B against A and C against A, but not B against C.

In the absence of direct evidence for the comparison of B against C, the adjusted indirect method provides a convenient way to conduct inferences on the relative effect of B versus C based on the point estimates of the relative effects of B versus A and C versus A and their associated standard errors. While these relative effects can be expressed on any suitable way for the data produced by the trials of B versus A and C versus A, we briefly explain below how the method works for the case where these data are binary in nature and the relative effects are expressed on the odds ratio scale.

Let 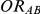, 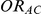 and 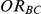 represent the true relative effects of B versus A, C versus A and B versus C, respectively. Furthermore, let 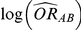 be the direct estimate of 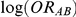 and 
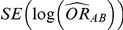
 be its associated estimated standard error, both of which are obtained via standard meta-analytic methods on the basis of the trials comparing A and B head-to-head. Similarly, let 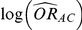 be the direct estimate of 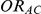 and 
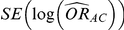
 be its corresponding estimated standard error, derived on the basis of standard meta-analytic methods from the trials comparing A and C directly.

According to the Bucher method, the indirect estimate of 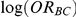 and its accompanying standard error can be obtained as:
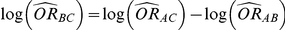


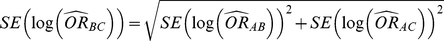



Combining these two pieces of information yields a 95% confidence interval for 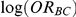:
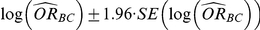



Exponentiation of the first and third of the above equations affords the derivation of point and confidence interval estimates of 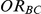. Specifically, the point estimate of 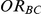 is given by
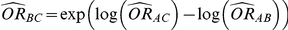



while the 95% confidence interval estimate of 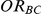 has end points given by
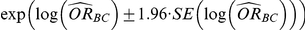



The 95% confidence interval for 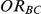 produced by the Bucher method can be used to test the null hypothesis 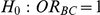 versus 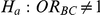. If this interval precludes the value 1 (which denotes a null relative effect of B compared to C), we reject the null hypothesis and conclude that the effect of B is significantly different from that of C (based on two-sided *α* = 5%). However, if this interval includes the value 1, we fail to reject the null hypothesis and conclude that the data do not provide sufficient evidence in favour of a difference in the effects of the two treatments.

In practice, the use of random-effects meta-analysis is typically recommended for deriving both (i) 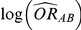 and 
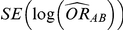
 and (ii) 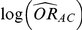 and 
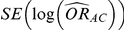
.

## Methods

### Generation of simulated data

Our simulation study was geared at the indirect comparison of two drugs B and C, which were compared head-to-head against another drug A, but not against each other. In this study, the direct comparisons of B versus A and C versus A were performed on the basis of trials with a binary outcome for each trial participant (i.e., participant experienced/did not experience the event of interest). For this reason, the true relative effects of B versus A and C versus A were expressed on the odds ratio scale as 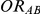 and 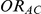. Similarly, the true relative effect of B versus C, which was of primary interest, was expressed on the odds ratio scale as 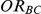.

Using Bucher's adjusted indirect comparison as a basis for conducting inferences on 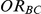, the simulation study was concerned with answering the following questions:

(I) What is the risk of over-estimation associated with the point estimation of 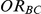
?

(II) What are the coverage properties of the confidence interval estimation method of 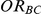
?

(III) What are the Type I error properties of the test of hypotheses 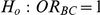 (null relative effect of C versus B) versus 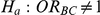 (non-null relative effect of C vs. B)?

(IV) What are the power properties of the test of hypotheses 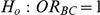 versus 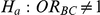
?

The simulation study included six different factors but was not set up as a full factorial experiment. These factors were: (1) 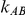, the number of trials pertaining to the B versus A comparison; (2) 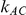, the number of trials pertaining to the C versus A comparison; (3) 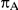, the true average event rate in the common comparator group A; (4) 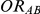, the true relative effect of B versus A, quantified as an odds ratio; (5) 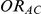, the true relative effect of C versus A, quantified as an odds ratio; (6) 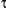, the between-study standard deviation, assumed constant across the comparisons B versus A and C versus A.

Given these factors, we explored the extent to which the performance of the indirect inference on 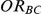 would be influenced by the size of 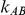 and 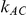, especially in situations where 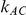 would either be equal to 1 or larger than 1 but much smaller than 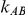. However, we also considered the influence of the remaining factors on the indirect inference on 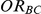.

In view of the above, we focused our attention on a limited number of combination of values for the factors 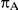, 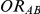 and 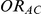, while allowing 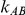 to take on the values 5, 10, 25 and 100, 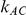 to take on the values 1 and 5, and the heterogeneity parameter 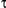 to take the following values: 0.001 (small between-study heterogeneity), 0.2 (moderate between-study heterogeneity) and 0.4 (large between-study heterogeneity). The combinations of values entertained for 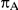, 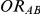 and 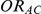 are listed in Table 1. Given any such combination of values, the resulting simulation experiment had a factorial structure with respect to the remaining factors 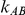, 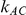 and 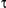.

**Table 1:** Combination of values for three of the parameters included in the simulation study, namely 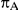, 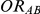 and 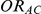, along with corresponding values of 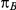 and 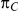.

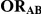	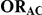	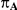	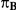	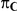
1.4	1.4	10%	13%	13%
1.4	1.4	30%	37%	37%
1.2	1.4	10%	12%	13%
1.2	1.4	30%	34%	38%
0.65	0.75	40%	30%	33%

We note the following in connection with the combination of values reported in Table 1 (See Table 1).

Knowing 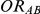 and 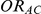 allows the determination of 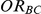, the true relative effect of B versus C, via the formula 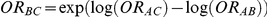. Using this formula, we can see that: (i) 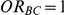 for those simulation settings where 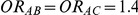; (ii) 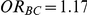 for those simulation settings where 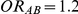 and 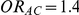 and (iii) 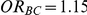 for those simulation settings where 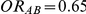 and 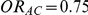.

In addition, if 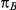 and 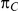
denote the true average event rates in groups B and C, respectively, we can determine the value of the former parameter from the values of 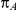 and 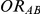 and that of the latter parameter from the values of 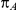 and 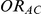: 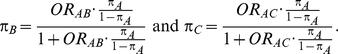



Table 1 shows the resulting values of 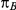 and 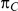 corresponding to the combinations of values of 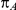, 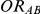 and 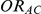 given in Table 1 (See Table 1). Based on Table 1, we see that the simulation settings for which 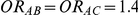 have equal true average event rates in groups B and C and that both of these rates are higher than the true average event rate in group A. Simulation settings for which 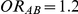 and 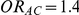 have different true average event rates in groups B and C (with the event rate in group C being slightly higher than that in group B). Both of these rates are higher than the true average event rate in the common comparator group A. Simulation settings for which 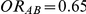 and 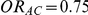 have a higher average event rate in group C than in group B, with both of these rates being smaller than the average event rate in group A.

For each combination of values for the six factors included in the simulation study, we generated 5,000 sets of 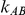 trials comparing B versus A and 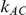 trials comparing C versus A and used them as input for conducting indirect inferences on the true relative effect of B versus C. The data for each of the 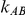 trials consisted of counts of events and number of participants in arms A and B of that trial. Similarly, the data for each of the 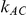 trials consisted of counts of events and number of participants in arms A and C of that trial. For simplicity, we discuss below only the generation of data from trials comparing B versus A.

Consider the j-th trial comparing B versus A amongst the 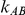 trials available for this comparison. The data for this trial were generated from the following model:
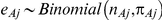


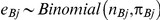


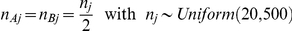


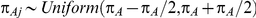


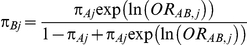


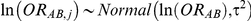



Here, 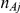 and 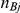 represent the number of participants in arms A and B of the 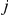-th trial comparing B versus A. Under the assumption of equal numbers of participants in both arms (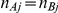), the total number of participants in the two arms was determined by sampling an integer between 20 and 500 participants.

The number of observed events in group A, 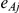, was drawn from a binomial distribution with parameters 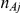 and 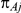, with 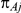 denoting the trial specific event rate in group A. The parameter 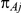 was drawn from a uniform distribution with support given by 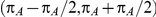, where 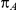 is the true average event rate in group A.

The observed number of events in arm B of the 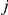-th trial comparing B versus A, 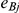, was drawn from a binomial distribution with parameters 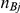 and 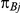, with 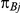 denoting the trial specific event rate in group B. The value of the latter parameter was derived on the basis of 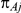 (trial specific event rate in group A) and 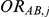 (trial-specific true relative effect of B versus A, expressed as an odds ratio). The natural logarithm of 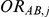 was sampled from a normal distribution with mean given by 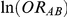 and variance given by 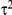, where 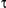 is the between-study standard deviation. The latter specification is consistent with assuming that the relative effects of B versus C are different across trials yet similar enough to be sampled from a common distribution.

Given the data 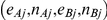, 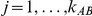, generated for the 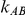 trials comparing B versus A, a random-effects meta-analysis based on the DerSimonian and Laird method was used to estimate 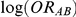 and its associated standard error.[5] These estimates – along with similarly obtained estimates of 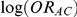 and its corresponding standard error - were used as inputs for the adjusted indirect comparisons method of Bucher.

### Measures of performance

The following measures of performance of the indirect inference on 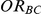 were considered in our simulation study:

- Risk of over-estimation;
- Confidence interval coverage;
- Type I error;
- Statistical power.

The risk of overestimation was evaluated only for those simulation settings where (i) 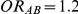 and 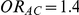 (hence 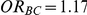) or (ii) 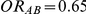 and 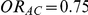 (hence 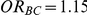). Given a simulation setting, this risk was computed by recording the proportion of times the indirect estimate of 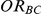 exceeded four different thresholds in the 5,000 simulations. The thresholds were selected to represent approximately a 20%, 30%, 50% and 75% increase in the true value of 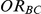. Specifically, when 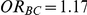, the thresholds were taken to be 1.40, 1.52, 1.75 and 2.05, respectively. When 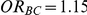, the thresholds were taken to be 1.38, 1.49, 1.72 and 2.01, respectively.

The confidence interval coverage was assessed for all simulation settings. Given a setting, coverage was evaluated by recording the percentage of simulations out of 5,000 for which the 95% confidence interval of 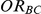 included the true value of 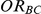
corresponding to that setting.

The type I error of the test of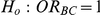 against 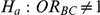 was evaluated only for those simulation settings with 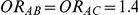 for which the null hypothesis was true (i.e., 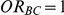). For each such setting, Type I error was assessed by tracking the percentage of simulations out of 5,000 which produced 95% confidence intervals for 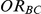 that excluded the value 1.

The statistical power of the test of 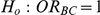 against 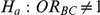 was computed only for those simulation settings with 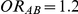 and 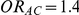 or 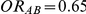 and 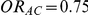, for which the null hypothesis was false. For each such setting, power was expressed as the percentage of simulations out of 5,000 which produced 95% confidence intervals for 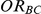 that excluded the value 1.

### Software Implementation

All simulations were performed using the freely available software package R 2.11.0.[6] All random-effects meta-analyses pertaining to the direct comparisons of B against A and C against A were conducted using the R package *metafor* (version 1.1-0).

## Results

### Risk of over-estimation

Table 2 presents the risk of over-estimation of 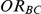 for simulation settings where 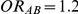 and 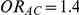 while Table 3 presents the same quantity for those settings where 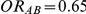 and 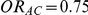.

**Table 2:** Percentage of simulations producing indirect estimates of 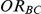 exceeding a given threshold corresponding to the simulation settings where 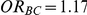.

			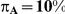			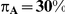		
		Threshold for judging over-estimation of 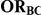	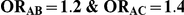			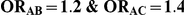		
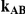	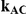		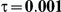	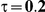	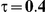	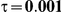	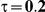	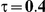
5	1	1.40	36.98	35.98	38.34	28.66	32.70	36.72
		1.52	31.00	30.86	34.04	21.04	25.96	31.34
		1.75	21.90	22.08	27.00	11.44	16.44	23.14
		2.05	14.38	14.56	19.98	6.04	9.36	15.40
10	1	1.40	35.30	36.56	37.58	27.92	31.58	34.88
		1.52	29.58	30.30	32.90	19.84	24.22	29.88
		1.75	21.54	22.66	26.22	10.58	14.74	22.20
		2.05	14.52	15.14	18.80	5.72	7.96	15.32
25	1	1.40	34.38	36.22	37.92	26.06	30.86	36.18
		1.52	27.72	30.44	33.18	18.18	23.94	30.84
		1.75	19.26	21.70	25.28	10.60	14.40	21.78
		2.05	12.56	14.46	18.10	5.40	7.58	13.96
100	1	1.40	34.12	35.16	37.18	26.02	31.24	36.70
		1.52	27.90	29.62	32.50	19.22	23.82	31.06
		1.75	19.94	21.12	25.12	10.16	14.30	22.76
		2.05	12.90	13.74	18.1	5.18	8.04	14.42
5	5	1.40	24.68	26.18	30.20	15.30	19.74	28.20
		1.52	16.34	18.08	23.24	7.16	11.26	20.52
		1.75	6.26	8.54	13.92	1.44	3.58	10.32
		2.05	2.12	3.04	7.14	0.18	0.68	4.34
10	5	1.40	22.00	22.88	28.78	11.36	16.56	24.98
		1.52	12.38	14.54	20.66	4.22	8.22	16.86
		1.75	4.24	5.66	10.28	0.54	2.16	6.78
		2.05	0.88	1.18	3.84	0.00	0.26	1.90
25	5	1.40	18.52	19.56	25.16	13.46	13.46	22.88
		1.52	9.58	11.24	17.46	5.78	5.78	14.28
		1.75	2.34	3.50	7.98	0.84	0.84	4.96
		2.05	0.30	0.62	2.8	0.08	0.08	1.40
100	5	1.40	17.16	18.40	23.58	6.62	8.76	20.66
		1.52	8.90	9.84	14.78	1.80	2.56	11.92
		1.75	2.22	2.36	5.92	0.12	0.06	4.20
		2.05	0.34	0.36	1.62	0.00	0.06	0.66

Four different thresholds were considered for each simulation setting: 1.40, 1.52, 1.75 and 2.05. These thresholds were chosen to represent an approximate increase of 20%, 30%, 50% and 75% in the value of 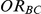. Reported percentages quantify the degree to which Bucher's method over-estimates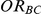. (Note: The true average event rate in group A was either 10% or 30%).

**Table 3:** Percentage of simulations producing indirect estimates of 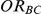 exceeding a given threshold corresponding to the simulation settings where 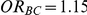 (or, equivalently, 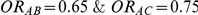).

			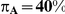		
		Threshold for judging over-estimation of 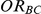	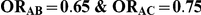		
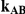	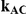		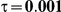	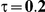	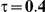
5	1	1.38	28.48	30.98	37.4
		1.49	21.12	24.92	32.04
		1.72	11.54	15.68	23.78
		2.01	5.54	8.78	15.92
10	1	1.38	26.46	31.56	34.72
		1.49	19.24	25.12	29.64
		1.72	10.26	14.52	21.86
		2.01	5.44	7.46	14.38
25	1	1.38	27.26	30.00	35.78
		1.49	20.12	23.58	30.52
		1.72	9.98	13.66	22.20
		2.01	5.36	7.10	14.34
100	1	1.38	26.12	30.00	35.76
		1.49	19.46	23.84	30.18
		1.72	9.52	14.00	21.18
		2.01	4.92	8.22	13.46
5	5	1.38	15.3	20.72	28.64
		1.49	7.88	12.50	21.06
		1.72	1.24	3.24	11.18
		2.01	0.10	0.66	4.64
10	5	1.38	11.46	17.42	25.04
		1.49	4.62	9.28	17.46
		1.72	0.44	2.20	7.70
		2.01	0.04	0.32	2.16
25	5	1.38	8.68	14.64	22.76
		1.49	2.58	7.04	14.44
		1.72	0.10	1.18	4.82
		2.01	0.00	0.06	1.04
100	5	1.38	16.84	12.14	21.16
		1.49	2.26	5.02	12.94
		1.72	0.02	0.84	4.42
		2.01	0.00	0.04	0.88

Four different thresholds were considered for each simulation setting: 1.38, 1.49, 1.72 and 2.01. These thresholds were chosen to represent an approximate increase of 20%, 30%, 50% and 75% in the value of 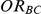. Reported percentages quantify the degree to which Bucher's method over-estimates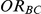. (Note: The true average event rate in group A was 40%).

When 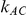 is 1, the true relative effect 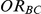 is often considerably overestimated. When 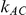 is 5, the overestimation is both less frequent and pronounced. For both of these values of 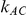, the more trials are available for the direct comparison of B versus A (i.e., the larger 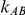), the smaller the risk of overestimation becomes.

### Coverage

Tables 4, 5 and 6 present the empirical coverage of the 95% confidence interval estimation method of Bucher for 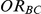 for simulation settings where 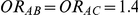, 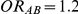 and 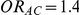, and 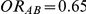 and 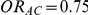, respectively. (The nominal coverage is 95%.)

**Table 4:** Coverage of the 95% confidence interval estimation method of Bucher for 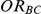.

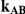	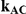	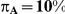			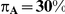		
		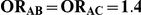			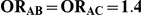		
		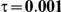	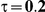	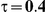	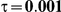	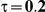	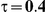
5	1	96.02	94.28	87.90	95.44	89.94	81.10
10	1	96.06	94.00	87.78	95.68	90.38	79.52
25	1	96.20	93.70	86.98	95.42	89.70	77.04
100	1	96.18	93.40	85.84	95.12	88.88	76.24
5	5	95.24	93.20	91.70	95.02	92.86	91.22
10	5	96.00	95.22	92.54	95.90	93.96	92.26
25	5	96.72	95.22	92.50	96.90	93.28	91.32
100	5	96.68	94.80	92.50	96.88	93.44	90.44

For each simulation setting, coverage was assessed by tracking the percentage of simulations producing confidence intervals for 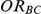 that captured the true value of 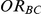. For settings where 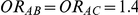, the true value of 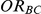
was 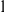. (Note: The true average event rate in group A was either 10% or 30%).

**Table 5:** Coverage of the 95% confidence interval estimation method of Bucher for 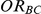.

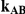	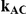	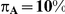			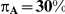		
		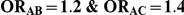			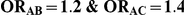		
		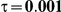	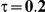	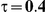	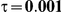	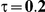	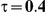
5	1	95.86	92.44	88.12	95.34	90.74	81.80
10	1	96.20	93.82	86.80	95.42	90.14	78.44
25	1	96.68	93.26	86.12	94.92	90.32	77.20
100	1	95.80	92.84	86.06	95.50	88.58	74.16
5	5	95.08	93.74	91.74	95.08	92.58	91.36
10	5	96.28	95.04	92.70	96.00	93.76	92.12
25	5	96.80	94.60	92.24	96.06	93.62	90.66
100	5	97.04	95.28	90.88	97.30	93.00	89.50

For each simulation setting, coverage was assessed by tracking the percentage of simulations producing confidence intervals for 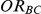 that captured the true value of 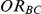. For settings where 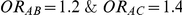, the true value of 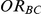 was 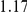. (Note: The true average event rate in group A was either 10% or 30%).

**Table 6:** Coverage of the 95% confidence interval estimation method of Bucher for 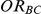.

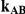	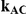	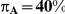		
		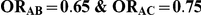		
		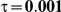	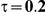	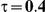
5	1	94.94	89.12	80.34
10	1	94.70	89.12	78.58
25	1	95.72	89.14	75.82
100	1	95.30	87.78	75.66
5	5	95.28	93.00	91.10
10	5	95.92	93.22	91.64
25	5	96.70	93.02	90.72
100	5	96.72	93.00	89.70

For each simulation setting, coverage was assessed by tracking the percentage of simulations producing confidence intervals for 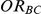 that captured the true value of 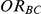. For settings where 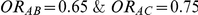, the true value of 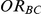 was 1.15. (Note: The true average event rate for group A was 40%).

For the all of these settings, the Bucher confidence interval estimation method generally reports empirical coverage values below the nominal coverage when the between-study heterogeneity is moderate or large (i.e., 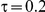 or 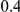) - a phenomenon referred to as undercoverage. As anticipated, the undercoverage tends to be more pronounced when 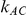 equals 1 than when 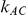 equals 5. Undercoverage could either be due to bias in the estimates of 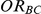 or due to underestimation of 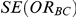
(which would cause the confidence interval to be artificially narrow).

When the between-study heterogeneity is small (i.e., 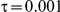), the estimated coverage of is generally either greater or slightly smaller than the nominal coverage, suggesting that the method produces conservative or valid confidence intervals. In particular, coverage exceeding the nominal level indicates that the Bucher method produces overly wide confidence intervals in this scenario. Increasing the value of 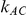 from 1 to 5 while keeping 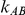 fixed has a minimal impact on the empirical coverage of the method. When the between-study heterogeneity is moderate (i.e., 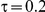) and especially large (i.e., 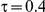), the estimated coverage is generally smaller than the nominal level. Increasing the value of 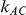 from 1 to 5 while keeping 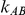 fixed results in coverages closer to the nominal level, albeit still off by as much as 5%.

### Type I Error

Table 7 displays the estimated Type I error associated with the test of 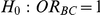 versus 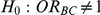 for those simulation settings with 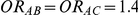. (The nominal Type I error is 5%.) For these settings, the estimated Type I error falls below the nominal Type I error when the between-study heterogeneity is small (i.e., 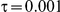) but exceeds the nominal Type I error when the between-study heterogeneity is moderate or large (i.e., 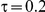
or 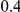). For fixed values of 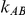 and 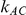, the levels of the estimated Type I error increases as the between-study heterogeneity increases. These findings hold for most values of 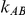 and regardless of whether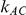 equals 1 or 5.

**Table 7:** Type I error associated with the test of the hypotheses 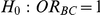 versus 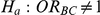.

		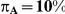			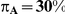		
		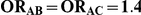			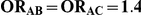		
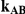	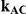	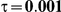	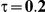	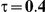	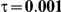	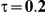	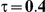
5	1	3.98	5.72	12.10	4.56	10.06	18.90
10	1	3.94	6.00	12.22	4.32	9.62	20.48
25	1	3.80	6.30	13.02	4.58	10.30	22.96
100	1	3.82	6.60	14.16	4.88	11.12	23.76
5	5	4.76	6.80	8.30	4.98	7.14	8.78
10	5	4.00	7.78	7.46	4.10	6.04	7.74
25	5	3.28	4.78	7.50	3.10	6.72	8.68
100	5	3.32	5.20	7.50	3.12	6.06	9.56

For each simulation setting where 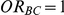 (or, equivalently, 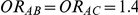), Type I error was assessed by tracking the percentage of simulations that produced 95% confidence intervals that excluded the value 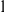. (Note: The true average event rate in group A was either 10% or 30%).

### Power

Tables 8 and 9 show the estimated power of the test of 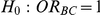 *versus* 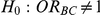 for those simulation settings with either 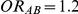 and 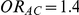, or 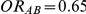 and 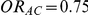. The results in these two tables show that this test is profoundly underpowered across both types of simulation settings. As expected, when 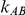 is kept fixed, increasing the value of 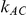 from 1 to 5 does result in an increase in the level of power, with the magnitude of this increase depending on the value of the between-study standard deviation 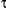. Similarly, when 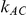 is kept fixed, increasing the value of 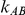 results in an increase in the level of power. Nevertheless, these increases in power are not large enough to overcome the issue of lack of power.

**Table 8:** Power associated with the test of the hypotheses 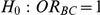 versus 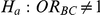.

		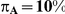			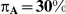		
		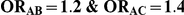			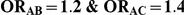		
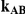	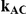	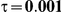	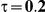	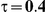	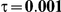	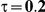	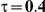
5	1	6.06	7.56	13.04	7.06	12.16	19.60
10	1	5.60	7.58	13.70	8.18	12.70	22.38
25	1	4.88	8.12	14.94	8.50	13.46	24.62
100	1	5.60	8.18	15.54	7.94	14.42	27.14
5	5	8.38	9.54	9.76	13.04	12.32	11.20
10	5	8.76	9.04	10.22	14.08	12.94	11.12
25	5	9.38	9.82	11.54	15.58	15.36	14.64
100	5	10.42	10.76	12.98	16.60	17.74	14.84

For each simulation setting where 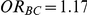 (or, equivalently, 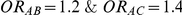), power was assessed by tracking the percentage of simulations that produced 95% confidence intervals for 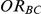 that excluded the value 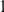. (Note: The true average event rate in group A was either 10% or 30%).

**Table 9:** Power associated with the test of the hypotheses 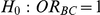 versus 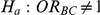.

		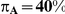		
		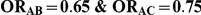		
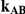	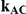	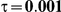	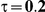	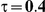
5	1	7.80	12.96	21.16
10	1	7.42	13.00	22.92
25	1	8.08	13.06	24.76
100	1	8.36	15.02	26.18
5	5	12.42	12.12	11.40
10	5	13.14	12.98	12.40
25	5	14.18	14.86	13.68
100	5	16.84	15.96	15.04

For each simulation setting where 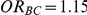 (or, equivalently, 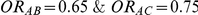), power was assessed by tracking the percentage of simulations that produced 95% confidence intervals for 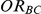 that excluded the value 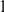. (Note: The true average event rate in group A was 40%).

## Discussion

Our study demonstrates that adjusted indirect comparisons are severely affected by the power and fragility of the data in the contributing comparisons. Given the growing acceptance and increased use of indirect comparisons in health-care decision-making, there is a need for caution when determining the strength of evidence from indirect comparisons. In particular, health-care decision makers should carefully assess the strength of evidence within each comparison (e.g., A vs B and A vs C) to grasp the reliability of the produced indirect point estimate and confidence interval.[7]

There are strengths and limitations to consider when interpreting our simulation study. Strengths of this study include the use of clinically reasonable assumptions about treatment effects and the simulation of varying scenarios of clinical importance versus statistical importance. Further, we explored inferential properties for the simplest form of indirect comparison (A vs B and A vs C). Such comparisons are present in multitude in more complex indirect comparisons and multiple treatment comparisons (MTC). To a considerable extent, our results may therefore extrapolate beyond the simulated scenarios as the underlying statistical assumptions used in MTC are similar.[8], [9] The limitations of our study include the overarching issue that we used simulations rather than real data for our analysis. We investigated the impact of the number of direct comparison trials on various statistical properties of an indirect comparison while allowing the sample size of each direct trial to follow a uniform distribution from 20 to 500. This setup ensured that our simulation scenarios are representative of real-world meta-analytic situations, where trials pertaining to a direct comparison typically vary in their sample sizes. However, our ability to reproduce such situations came with a price: we were unable to assess the effect of the trial sample size on the power of an indirect comparison, due to its confounding with the other factors examined in our simulation study, such as heterogeneity. Furthermore, we assessed the risk of overestimation, confidence interval coverage and statistical power of an indirect comparison involving two treatments, but we did not examine these statistical features for the direct comparison involving the same two treatments. One reason for this is that, in practice, indirect comparisons are performed specifically when direct comparisons cannot be performed due to a lack of direct evidence. While it is possible to expand our simulation study to include a comparison of the statistical properties of direct and indirect comparison concerning the same treatments, we chose not to pursue this here in an effort to preserve the simplicity of our findings and interpretations. We hope to address this issue in a future paper. We used the DerSimonian-Laird random-effects model which makes use of the DerSimonian-Laird estimator to estimate the between-study variation. This estimator has been known to underestimate the between-study variance.[10 11, 12]Thus, the undercoverage and inflation of type I error we detected in simulation scenarios with moderate or large heterogeneity may in part be caused by properties of this estimator rather than properties of the Bucher adjusted indirect comparison method. [1]

The use of indirect comparisons and MTC analyses is growing in popularity in both journal publications and by health technology assessments.[2] The criticisms of both approaches is that it is not obvious where biases or errors may arise from, including issues of individual trial bias, trial-level differences across comparisons, and problems in the conduct of the indirect model.[13] Authors and readers appear to have difficulty interpreting the quality of indirect comparison meta-analysis and tools for critical appraisal do not yet exist.[14] Our study demonstrates that caution is warranted, especially in situations where low numbers of trials are included in any treatment arm. Insights from empirical studies are crucially needed to further inform this issue. Further, we hope investigate the fragility and power associated with point estimation and hypothesis testing in MTC in a near future.

In conclusion, indirect comparisons with 1 or 5 trials in one of the indirect comparison arms are consistently underpowered (power <20%), regardless of the number of trials in the other indirect comparison arm. Results from indirect comparisons may especially become unreliable with the heterogeneity is moderate or large. Authors and readers of indirect comparisons should exercise caution and scepticism when interpreting results from indirect comparisons.
